# Association between chronic pain and pre-frailty in Japanese community-dwelling older adults: A cross-sectional study

**DOI:** 10.1371/journal.pone.0236111

**Published:** 2020-08-13

**Authors:** Ryota Imai, Masakazu Imaoka, Hidetoshi Nakao, Mitsumasa Hida, Fumie Tazaki, Tomoko Omizu, Tomoya Ishigaki, Misa Nakamura

**Affiliations:** 1 School of Rehabilitation, Osaka Kawasaki Rehabilitation University, Kaizuka, Osaka, Japan; 2 Department of Rehabilitation, Kansai University of Welfare Sciences, Kashihara, Osaka, Japan; 3 Department of Physical Therapy, Faculty of Rehabilitation Sciences, Nagoya Gakuin University, Nagoya, Aichi, Japan; Ritsumeikan University, JAPAN

## Abstract

A relationship between chronic pain and frailty has been reported. The early detection and prevention of frailty are recommended, in part because community-dwelling older adults in a pre-frailty state may return to a healthy state. The relationship between chronic pain and pre-frailty is not known. Toward the goal of promoting a reversible return to health from pre-frailty, we investigated the relationship between chronic pain and pre-frailty among community-dwelling older adults. We assessed the frailty and chronic pain of 107 older adults who were participating in community health checks. The status of physical frailty was based on the five components described by Fried (2001): muscle weakness shown by handgrip strength, slowness of gait speed, weight loss, low physical activity, and exhaustion. Chronic pain was assessed based on pain intensity, the Pain Catastrophizing Scale (PCS), the Japanese version of the Geriatric Depression Scale-15 (GDS-15), and the Central Sensitization Inventory (CSI). The prevalence of chronic pain with pre-frailty was 40.2%. A hierarchical analysis revealed that PCS-measured helplessness (odds ratio [OR]: 0.88) and the CSI (OR: 0.87) were significant factors associated with the presence of chronic pain with pre-frailty. The prevalence of chronic pain with pre-frailty was high, and chronic pain and pre-frailty were strongly related. New intervention or prevention programs that take into account both chronic pain and pre-frailty must be created as soon as possible.

## Introduction

Chronic pain is common among community-dwelling older adults. An epidemiological study indicated that the proportion of community-dwelling older adults with chronic pain is 65.0%–78.8% [1]. The prevalence of pain is also high in general populations and increases with age. In studies of community-dwelling older adults, the prevalence of pain was 40%, and that in institutionalized individuals was 80% [2–5]. Chronic pain increases healthcare costs and decreases the quality of life of those who experience it [6]. In addition, the presence of chronic pain is associated with a decline in activities of daily living (ADLs) because of the body's deteriorating physical function [7], a poor psychological status [8], and low physical activity levels [9]. In Japan, the number of community-dwelling older adults who require long-term care due to physical deterioration has increased because of the rapid aging of the population. An effective approach to pain management is thus needed for community-dwelling older adults with chronic pain.

Wade et al. (2016) reported that in older adults, the presence of chronic pain was associated with an increased risk of frailty [10]. With advancing age, the prevalence of pain and that of frailty both increase considerably, and both pain and frailty are associated with functional decline and have negative impacts on older adults' quality of life. Hirase et al. (2017) reported that for community-dwelling older adults with frailty in Japan, chronic pain can negatively influence sensory, emotional, and cognitive aspects of pain, leading to a decline in ADLs and lower physical activity [11]. A full pain assessment focusing on sensory and emotional aspects of pain is important to identify frailty among older adults. Persistent pain was shown to be a risk factor for the development of frailty [12]. It has been suggested that pain should be incorporated into the criteria used to define frailty, given that pain may be an additional manifestation of the pathophysiological changes of frailty [13]. The diagnosis of frailty is thus important for providing effective interventions for community-dwelling older adults with chronic pain.

The early detection and prevention of frailty are recommended for community-dwelling older adults with a pre-frailty status, as they may be able to return to a healthy state. We hypothesized that interventions for pre-frailty combined with pain management may help improve both pre-frailty and chronic pain. Among community-dwelling older adults, the rate of frailty is low (5%–8%) but the rate of pre-frailty is high (40%–50%) [14]. Because pre-frailty poses a higher risk of needing the support and care certified by Japan's long-term care insurance system, interventions for pre-frailty are needed [15]. The relationship between pre-frailty and chronic pain remains to be established; doing so will contribute to new intervention and prevention programs for adults with a pre-frailty status. We conducted the present study to clarify the relationship between pre-frailty and chronic pain among community-dwelling older adults, toward the goal of helping these individuals return to normalcy from pre-frailty.

## Subjects and methods

### Design and subjects

This cross-sectional study was conducted in the city of Kaizuka, Japan from 17 August to 12 September 2019. The recruitment period of a community health check-up program was from 1 July to 8 August 2019. There were three recruitment criteria for participation: (1) living in Kaizuka City, (2) age ≥40 years, (3) with no physician-ordered exercise ban. We published a flyer for the community health check-up program in local newspapers and placed the flyer in public offices such as the city hall.

A community health check-up program expected 325 people to attend, and 54 did not; thus, a total of 275 older adults participated in the program. The inclusion criteria were as follows: age ≥65 years, living at home, able to walk outdoors, and performing ADLs independently. Three community centers in Kaizuka were used to conduct the community health check-up program. Individuals who were unable to respond to questions because of cognitive impairment were excluded. Well-trained volunteer staff and students affiliated with medical colleges performed all of the assessments of the subjects.

This study was approved by the Osaka Kawasaki Rehabilitation University (OKRU-A016) and conformed to the guidelines of the 2008 Helsinki Declaration of Human Rights. Written informed consent to participate was obtained from each subject before the study started.

### Body composition

Each subject's body mass index (BMI) was calculated by dividing the body weight (kg) by height squared (m^2^). The appendicular skeletal muscle mass index (SMI) was derived from the appendicular muscle mass (kg) divided by height squared (m^2^) using a bioelectrical impedance device (InBody270; InBody, Tokyo). The body fat percentage was also measured using its device.

### Frailty assessment

The status of physical frailty was based on the presence of the five components reported by Fried: muscle weakness shown by handgrip strength, slowness of gait speed, weight loss, low physical activity, and exhaustion [16, 17]. Handgrip strength is a well-known measure of muscle strength and is significantly associated with whole-body muscle strength. The maximum voluntary isometric strength of each of the present subjects' handgrip was measured with the subject's dominant hand while he or she was in a standing position, by means of a hand dynamometer (Grip-D, Takei, Niigata, Japan). Weakness was defined using the maximum grip strength by the cutoff values of <26 kg for men and <18 kg for women [10]. Other bodily movements were not permitted during the measurement. The skeletal muscle mass index (SMI) was measured by using the Inbody270 device.

For the gait speed assessment, the subject was instructed to walk 6.4 m (divided into two 2.0-m zones at each end and a 2.4-m middle zone) at a speed the subject found comfortable. The time needed (in sec) to pass the 2.4-m middle zone was measured for the calculation of gait speed (m/sec). The subject could use a cane or walker if he or she was unable to perform the gait test independently. The average of five gait tests' results was used for the evaluation. Slowness was defined using <1.0 m/sec as the normal walking speed cutoff value.

Weight loss was defined as a subject having lost >2 kg in the prior 6 months [18]. 'Low physical activity level' was defined by the subject's answers to the following questions: (1) "Do you perform light exercise or sports for your health?" and (2) "Do you regularly exercise or engage in a sport?" The subjects who answered 'no' to both questions were categorized as having a low activity level [19]. The subjects who did not have any of the five characteristics (handgrip strength, slowness of gait speed, weight loss, low physical activity, and exhaustion) were categorized as not physically frail; those who had one or two characteristics were categorized as physically pre-frail, and those who had three or more characteristics were categorized as physically frail [15, 20].

### Pain assessment

Pain was assessed by documenting the subject's pain sites and the intensity of his or her maximum pain with the use of a numerical rating scale (NRS) from 0 ("no pain") to 10 ("the worst imaginable pain"). In addition, the subjects were asked to describe the site(s) of their pain (e.g., neck, shoulder, upper limb, lumbar, hip and knee). We also assessed the presence of chronic pain, which was defined as related symptoms within the prior month that had continued for ≥3 months and corresponded to an NRS score of ≥1 at the site of maximum pain [21].

### Pain-related psychological assessment

We also administered the Pain Catastrophizing Scale (PCS) [22] and the Japanese version of the Geriatric Depression Scale-15 (GDS-15) [23] to each subject. The PCS is comprised of 13 items in three domains: rumination (five items), helplessness (five items), and magnification (three items). Each item is rated on a five-point scale from 0 ("not at all") to 4 ("all the time"). The total PCS score ranges from 0 to 52; higher scores indicate greater pain catastrophizing. The GDS-15 is a self-report evaluation comprised of 15 items: 10 items confirm depression if the answers are positive, and the remaining five items confirm depression if the answers are negative. Normal scores range from 0 to 4, depending on factors such as age, education, and complaints; a score of 5–8 indicates mild depression, 9–11 moderate depression, and 12–15 severe depression. In this study, the GDS-15 was administered with permission from the copyright holder.

The Central Sensitization Inventory (CSI) was developed as a screening tool to identify and quantify patients with central sensitization (CS)-related symptoms [24, 25]. The CSI-9 (a short version of the CSI) is a symptomatological and self-reported questionnaire consisting of nine items assessing health-related symptoms that are common among individuals with central sensitivity syndromes [26]. The validity of the CSI as an assessment tool for patients with chronic pain has been demonstrated, and the total score of the CSI is related to widespread pain, pain intensity, disability, quality of life (QOL), and pain catastrophizing [27].

### Statistical analyses

The number of male subjects was insufficient, and we therefore decided to analyze only the female subjects because all of the outcome measurements could be affected by the asymmetrical numbers of males and females. To determine the prevalence of chronic pain in the subjects, we divided the subjects into those with chronic pain (the chronic pain group, n = 84) and those without chronic pain (the non-chronic pain group, n = 117). The Kolmogorov-Smirnov test was used to confirm the normality of the distribution of each evaluation item. We used the χ^2^ test, unpaired *t*-test, and Mann-Whitney U-test to evaluate significant differences between the two subject groups. We conducted a hierarchical regression analysis to ascertain the factors most strongly associated with chronic pain with pre-frailty. The Model 1 regression used the subjects' basic information (i.e., gender, age, height, and weight), and Model 2 used that information plus the subjects' psychological factors of pain; that is, the PCS items (rumination, helplessness, magnification) and the GDS-15 score. Model 3 added the CSI value, and gait speed was entered in Model 4.

To further compare the chronic pain and non-pain groups, we calculated the proportions of subjects with frailty, pre-frailty, and non-frailty in each group: chronic pain with frailty, pre-frailty and non-frailty, and non-pain with frailty, pre-frailty and non-frailty. A one-way analysis of variance (ANOVA) for continuous measures was performed to assess differences between each pair of groups (chronic pain with pre-frailty, chronic pain with non-frailty, non-pain with pre-frailty, and non-pain non-frailty) in outcome measures, and post-hoc tests using Tukey's correction were then used. Differences in helplessness and the GDS-15 score were examined by the Kruskal-Wallis test. The analysis was performed in the same way as the hierarchical method described above to identify the factors associated with chronic pain with pre-frailty, and thus the Model 1 and 2 regressions used the same outcomes. For Model 3, we entered the CSI score in addition to the PCS (rumination, helplessness, magnification) and the GDS-15 score, and for Model 4, we entered the gait speed. Statistical analyses were conducted using SPSS ver. 22.0 (IBM, Armonk, NY). The significance level was set at p<0.05 for all statistical analyses.

## Results

**Fig 1:**
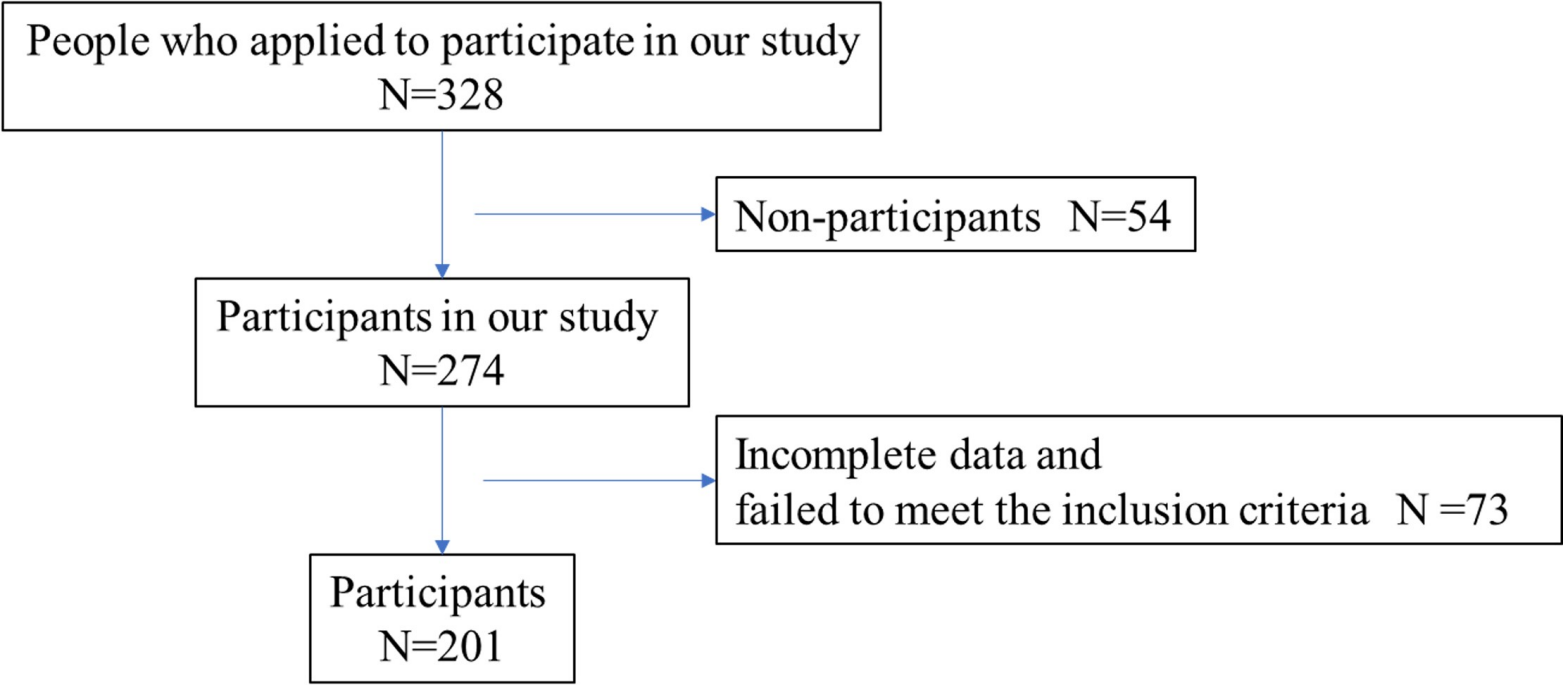
Flowchart of inclusion and exclusion criteria for this study.

Fig 1 outlines the study's recruitment of subjects. A total of 274 older adults were screened as potential subjects; 57 subjects did not complete all of the evaluations and another 16 failed to meet the inclusion criteria (n = 16). The final number of subjects for the analyses was 201 older adults.

### The characteristics of the chronic pain and non-chronic pain subjects

The characteristics of the chronic pain and non-chronic pain groups are summarized in Table 1. The proportion of chronic pain was approx. 41.8% (84 of the 201 subjects). There were no significant differences between the chronic pain and non-chronic pain groups in age, sex, height, body weight, BMI, body fat mass (BFM), SMI, or grip strength. Compared to the non-chronic pain group, the chronic pain group showed significantly poorer scores for pain intensity, rumination, PCS total score, rumination, helplessness, magnification, CSI and GDS-15 score (Table 1).

**Table 1:** Characteristic of the chronic pain group and the non-chronic pain group.

Characteristics	Total (n = 201)	Chronic pain (n = 84)	Non-chronic pain (n = 117)	p-value
Age	71.8 (8.8)	72.4(8.8)	71.5 (8.8)	n.s.
Height, cm	151.7 (5.9)	151.2 (5.6)	152.1 (6.0)	n.s.
Weight, kg	50.9 (8.1)	51.8 (8.3)	50.4 (7.9)	n.s.
BMI, kg/m^2^	22.1 (3.3)	22.6 (3.2)	22.6 (3.2)	n.s.
BFM	16.0 (5.6)	16.5 (5.9)	15.7 (5.3)	n.s.
SMI	5.7 (0.9)	5.7 (0.7)	5.6 (0.7)	n.s.
MMSE	29 (1.9)	29.1 (1.7)	28.9 (1.9)	n.s.
NRS at the site of maximum pain	3.2 (2.1)	3.8 (1.7)	0.9 (1.5)	<0.01
Site of pain, n (%)				
Neck	4 (2.0)	3 (3.6)	1 (0.9)	n.s.
Shoulder	32 (15.9)	26 (31.0)	6 (5.1)	<0.01
Upper limb	7 (3.5)	7 (8.3)	0 (0)	<0.01
Lumbar	47 (23.4)	41 (49.4)	6 (5.1)	<0.01
Hip	9 (3.9)	8 (9.5)	1 (0.9)	<0.01
Knee	43 (23.4)	38 (45.2)	5 (4.3)	<0.01
Under knee	24 (11.9)	20 (23.8)	4 (3.4)	<0.01
PCS total score	15.8 (11.7)	18.5 (9.3)	13.9 (12.8)	<0.01
Rumination	8.3 (5.5)	9.9 (4.5)	7.2 (5.9)	<0.01
Helplessness	4.4 (4.2)	5.1 (3.5)	3.9 (4.6)	<0.01
Magnification	3.1 (3.2)	3.5 (3.0)	2.7 (3.3)	<0.01
CSI	7.9 (3.2)	9.8 (6.3)	6.4 (4.8)	<0.01
GDS score	3.6 (2.8)	4.1 (3.2)	3.3 (2.5)	<0.05
Gait speed, m/s	1.29 (0.2)	1.25 (0.22)	1.31 (0.19)	<0.05
Grip strength, kg	21 (4.3)	20.1 (4.5)	21.1 (4.5)	n.s.

BFM: body fat mass, BMI: body mass index, CSI: Central Sensitization Inventory, GDS-15: Geriatric Depression Scale-15, n.s.: non-significant, PCS: Pain Catastrophizing Scale, SMI: skeletal muscle mass index.

### Hierarchical regression analysis for chronic pain

The results of the hierarchical regression analysis are presented in Table 2. Items demonstrating significant differences in previous between-group comparisons were designated as the independent variables. We evaluated multicollinearity among the independent variables using Pearson's correlation coefficient and Spearman's rank correlation coefficient.

**Table 2:** Hierarchical regression analysis identifying the factors associated with the presence of chronic pain.

Independent variables	OR	95% CI	p-value
*Model 1*			
Age	1	0.97–1.04	0.9
Height	0.81	0.56–1.17	0,27
Weight	1.32	0.77–2.27	0,31
BMI	0.56	0.16–1.98	0.37
*Model 2*			
Age	0.99	0.96–1.04	0.96
Height	0.8	0.55–1.17	0.25
Weight	1.35	0.77–2.35	0.29
BMI	0.54	0.15–1.98	0.36
Rumination	1.11	1.03–1.19	<0.01
Helplessness	0.97	0.88–1.01	0.54
GDS score	1.07	0.96–1.19	0.2
*Model 3*			
Age	1	0.98–1.08	0.69
Height	0.74	0.51–1.46	0.15
Weight	1.48	0.58–2.76	0.19
BMI	0.43	0.11–3.87	0.23
Rumination	1,09	0.94–1.14	0.03
Helplessness	0.97	0.88–1.19	0.48
GDS score	0.97	0.84–1.1	0.63
CSI	1.12	0.92–1.2	<0.01
*Model 4*			
Age	1	0.96–1.04	0.86
Height	0.73	0.49–1.09	0.13
Weight	1.53	0.85–2.77	0.16
BMI	0.39	0.1–1.56	0.19
Rumination	1.09	1.01–1.18	0.03
Helplessness	0.96	0.88–1.06	0.41
GDS score	0.97	0.86–1.1	0.66
CSI	1.11	1.04–1.19	<0.01
Gait speed (m/s)	0.45	0.85–2.4	0.35

BMI: body mass index, CSI: Central Sensitization Inventory, GDS-15: Geriatric Depression Scale-15.

There were strong correlations between the total PCS score and each of the three PCS domain scores (r>0.89). There was also a strong correlation between the PCS helplessness and magnification scores (r>0.81). We therefore excluded the PCS total and magnification scores as independent variables. Based on this analysis, PCS helplessness (odds ratio [OR]: 0.97) was identified as a significant factor associated with the presence of chronic pain in Model 2 (Table 2). In Model 3, CSI (OR: 1.12) was also identified as a significant factor associated with the presence of chronic pain. Although gait speed was added to Model 4, gait speed was not shown to be a significant factor.

### The characteristics of the subjects with both chronic pain and physical frailty

Table 3 summarizes the proportions of physically frail subjects with and without chronic pain and the comparisons of outcome measures. There was little frailty either with or without chronic pain (n = 9), so we analyzed four groups by a one-way ANOVA: chronic pain with pre-frailty (CPPF, n = 43), chronic pain with non-frailty (CPNF, n = 33), non-chronic pain with pre-frailty (NCPPF, n = 8), and non-chronic pain with non-frailty (NCPNF, n = 14).

**Table 3:** Group characteristics and comparisons of outcome measures.

	Chronic pain			Non-chronic pain		
	Frailty	Pre-frailty	Non-frailty	Frailty	Pre-frailty	Non-frailty
	(n = 8)	(n = 43)	(n = 33)	(n = 1)	(n = 8)	(n = 14)
Age	768.4(6.7)	71.8 (9.8)	71.7 (7.3)	72.0 (0)	74.1 (8.4)	71.3 (7.9)
Height	145.1 (3.1)	151.1 (5.9)	152.6 (4.7)	153.0 (0)	150.6 (5.4)	152.8 (5.2)
Weight	45.9 (6.7)	50.6 (7.2)	54.9 (9.1)	51.3 (0)	50.7 (5.3)	54.1 (8.7)
BMI	21.9 (3.4)	22.1 (2.9)	23.5 (4.3)	21.9 (0)	22.3 (1.6)	23.3 (4.3)
BFM	13.9 (6.5)	15.7 (5.2)	18.2 (6.4)	15.9 (0)	17.3 (3.4)	17.4 (6.3)
SMI	5.2 (0.5)	5.6 (0.6)	6.0 (0.7)	5.7 (0)	5.5 (0.6)	6.1 (0.8)
Pain intensity	5.3 (1.9)	4.3 (1.6)	2.9 (1.3)**	2.0 (0)	2.4 (1.5)**	1.4 (1.4)**^,^^#^
Site of pain, n (%)						
Neck	0 (0)	3 (7.0)	0 (0)	0 (0)	1 (12.5)	0 (0)
Shoulder	2 (25)	3 (7.0)	21 (63.6)	1 (100)	5 (62.5)	0 (0)
Upper limb	2 (25)	3 (7.0)	2 (6.0)	0 (0)	0 (0)	0 (0)
Lumbar	3 (37.5)	23 (53.5)	15 (45.5)	1 (100)	4 (50)	1 (7.1)
Hip	1 (12.5)	7 (16.3)	0 (0)	0 (0)	1 (12.5)	0 (0)
Knee	4 (50)	27 (62.8)	7 (21.2)	0 (0)	5 (62.5)	0 (0)
Under knee	1 (37.5)	19 (44.2)	0 (0)	0 (0)	4 (50)	0 (0)
PCS total score	21.5 (8.4)	21.0 (8.7)	14.5 (9.2)*	0	6.1 (4.2)**	10.4 (12.9)**
Rumination	11.4 (3.7)	10.8 (4.4)	8.4 (4.5)	0	3.4 (1.9)**^,#^	5.4 (5.8)**
Helplessness	8.4 (3.2)	6.0 (3.2)	3.6 (3.6)**	0	1.9 (2.0)**	2.6 (4.7)**
Magnification	3.8 (3.7)	4.3 (2.9)	2.5 (2.6)*	0	0.8 (1.6)*	2.4 (3.2)*
CSI	14.4 (4.9)	11.3 (7.2)	6.8 (3.3)*	10.0 (0)	7.9 (4.3)	6.3 (4.4)*
GDS score	6.3 (2.1)	4.7 (3.4)	5.1 (2.5)**	4.0 (0)	5.1 (2.5)^#^	3.5 (3.1)
Gait speed (m/s)	1.09 (0.3)	1.21 (0,22)	1.34 (0.17)*	1.39 (0)	1.26 (0.14)	1.34 (0.19)
Grip strength	15.0 (2.3)	21.0 (4.6)	22.3 (4.6)	16.4 (0)	19.2 (5.9)	21.7 (2.1)

*p<0.05 (vs. chronic pain with pre-frailty)

**p<0.01 (vs. chronic pain with pre-frailty)

^#^p<0.05 (vs. chronic pain with non-frailty). BMI: body mass index, BFM: body fat mass, CSI: Central Sensitization Inventory, GDS-15: Geriatric Depression Scale-15, SMI: skeletal muscle mass index.

There were no significant differences among these groups in age, sex, height, weight, BMI, SMI or BFM (Table 3). In the pain-related assessment, the CPPF group showed significantly higher pain intensity than the other three groups (p<0.01), and the pain intensity in the CPNF group was significantly higher than that in the NCNF group (p<0.05). The CPPF group's PCS total score was significantly higher than those of the other three groups (p<0.05). In addition, the helplessness and magnification scores in the CPPF group were significantly higher than those in the other three groups (p<0.05). The CPNF group's rumination score was significantly higher than that of the NCPNF (p<0.05) group. The CPPF subjects' GDS-15 scores and CSI scores were significantly different from only those of the CPNF subjects (p<0.05). In the physical assessment, there were no significant differences among the groups in grip strength, but the CPPF group's gait speed was significantly slower compared to that of the subjects with CPNF (p<0.05) (Table 3).

### Hierarchical analysis of the relationship between chronic pain and pre-frailty

The results of the logistic regression analysis are shown in Table 4. The analyses were conducted with the presence of CPPF as the dependent variable. As in the above-described analysis, the PCS total score and the PCS magnification score were excluded as independent variables. The results of this analysis identified PCS rumination (OR: 0.9) and helplessness (OR: 0.85) as significant factors associated with the presence of CPPF in Model 2. In Model 3, helplessness and CSI were shown to be significant. Finally, although gait speed was added to Model 4, gait speed was not selected as a significant factor associated with the presence of CPPF, but helplessness (OR: 0.88) and CSI (OR: 0.87) were identified as significant.

**Table 4:** Hierarchical regression analysis identifying the factors associated with the presence of chronic pain with pre-frailty.

Independent variables	OR	95%CI	p-value
*Model 1*			
Age	1.01	0.97–1.06	0.49
Height	1.27	0.78–2.08	0.34
Weight	0.77	0.39–1.52	0.44
BMI	2.11	0.41–10.77	0.37
*Model 2*			
Age	1.02	0.97–1.07	0.53
Height	1.31	0.74–2.31	0.35
Weight	0.75	0.34–1.63	0.47
BMI	2.31	0.36–14.8	0.38
Rumination	0.9	0.81–1.01	0.09
Helplessness	0.85	0.73–0.98	0.03
GDS score	0.86	0.73–1.01	0.06
*Model 3*			
Age	1.00	0.94–1.06	0.69
Height	1.63	0.88–2.99	0.15
Weight	0.57	0.25–1.23	0.19
BMI	4.6	0.64–33.1	0.23
Rumination	0.93	0.82–1.06	0.48
Helplessness	0.88	0.75–1.04	0.03
GDS score	0.94	0.78–1.13	0.63
CSI	0.85	0.75–0.96	0.01
*Model 4*			
Age	1.00	0.95–1.08	0.65
Height	1.62	0.87–3.03	0.13
Weight	0.55	0.24–1.28	0.16
BMI	5.15	0.68–38.9	0.11
Rumination	0.93	0.82–1.06	0.26
Helplessness	0.88	0.75–1.04	0.04
GDS score	0.90	0.74–1.10	0.29
CSI	0.87	0.76–0.98	0.03
Gait speed (m/s)	23.10	1.55–34.48	0.06

BMI: body mass index, CSI: Central Sensitization Inventory

GDS-15: Geriatric Depression Scale-15.

## Discussion

We sought to reveal the relationship between chronic pain and pre-frailty among community-dwelling older adults. In our subjects, the prevalence of chronic pain was 41.8%, and among all subjects, the prevalence of chronic pain with pre-frailty was 40.2%. It is thus necessary to assess both chronic pain and pre-frailty when we intervene for community-dwelling older adults.

We first determined the proportion of chronic pain among community-dwelling older adults living in a mid-sized city in Japan, and we assessed the characteristics of the subjects who reported experiencing chronic pain. The proportion of the subjects with chronic pain was approx. 41.8%, which is low to compared to epidemiological reports that the proportion of community-dwelling older adults with chronic pain is 65.0%–78.8% [1, 28]. Our present chronic pain group had significantly higher PCS total scores compared to the non-chronic pain group, as was observed in another study conducted in Japan [11]. The results of our hierarchical regression analysis revealed that the PCS domain 'helplessness' and the CSI were the most significant factors associated with the presence of chronic pain. Hirase et al. reported that the PCS helplessness scale was most closely associated with chronic pain in community-dwelling older adults [11]. Thus, helplessness of pain-related catastrophizing is the factor that is most strongly associated with the presence of chronic pain. When an individual's pain intensity persists for a long time, his or her sense of helplessness might therefore become high.

The CSI values for community-dwelling older adults had been unclear, but our results showed that the present community-dwelling older subjects with chronic pain had significantly greater CSI values compared to those who did not have chronic pain. Central sensitization is a proposed physiological phenomenon in which dysregulation in the central nervous system causes neuronal dysregulation and hyperexcitability, resulting in hypersensitivity to both noxious and non-noxious stimuli [24]. Central sensitization has been linked to CS syndromes including fibromyalgia, chronic fatigue syndrome, and temporomandibular disorder [29–31]. Central sensitization may also augment or amplify the symptom intensity in patients with musculoskeletal disorders such as chronic lower back, knee, or shoulder pain [32–34]. Several research groups have reported that preoperative CS may contribute to the development of postoperative chronic pain [35–37]. Since community-dwelling older adults with chronic pain can find it difficult to ease their pain, CS may be involved, and it is therefore important to evaluate CS in these individuals.

The association between pain and frailty was described in a systematic review [38]. Most of the studies in that review observed an association between chronic pain and frailty in terms of prevalence; approx. 40%–50% of frail patients had chronic pain [4, 39, 40]. Other studies described a positive relationship between chronic pain and frailty [41–43]. It is generally thought that compared to patients who are not experiencing chronic pain, patients with chronic pain are more likely to become frail. In a study with an 8-year-long follow-up, pain alone was reported to cause a fairly high percentage of frailty compared to the patients without pain [43].

There have been many investigations of pain and frailty, but to the best of our knowledge, few studies have examined the state of pre-frailty. In our present population, the prevalence of chronic pain with pre-frailty was 40.2% and the subjects with chronic pain along with pre-frailty not only had higher levels of pain-related factors than the subjects without these characteristics; they also had significantly different gait speeds and GDS-15 scores. Additionally, based on our hierarchical regression analysis, the PCS domain 'helplessness' and the CSI score were the most significant factors associated with the presence of chronic pain with pre-frailty, whereas the GDS-15 score was not selected.

The presence of chronic pain and chronic pain with pre-frailty was closely associated with the CSI in these community-dwelling older adults. Our present findings indicate that CSI and helplessness might be a significant factor associated with the presence of chronic pain with pre-frailty. In other words, in community-dwelling older adults we must assess and prevent both chronic pain and the pre-frailty status, rather than assessing and preventing pre-frailty alone or chronic pain alone.

Research on frailty and chronic pain has been conducted before, but the present study focused on pre-frailty. The likelihood of improving chronic pain and frailty is considered poor. The early detection and prevention of frailty among pre-frail community-dwelling older adults are recommended, as these may help such individuals return to a healthy state. Our present findings suggest that immediate intervention for CS and helplessness is needed in older adults with pre-frailty status and chronic pain. Significant improvement may be expected by using not only cognitive behavior therapy, pain neuroscience education and pain management, but also exercise intervention for pre-frail adults.

There are several limitations to the present study. Compared to previous studies' subjects, our subjects had less pain, and the prevalence of frailty and that of chronic pain with frailty were low. There were also no significant between-group differences in motor function or GDS-15 scores. Thus, compared to all individuals with chronic pain, our subjects' symptoms might not be as severe.

## Conclusions

We investigated the relationship between chronic pain and the pre-frailty status among community-dwelling older adults in a city in Japan. The prevalence of chronic pain with pre-frailty was high, and thus new interventions or prevention programs that take into account both chronic pain and pre-frailty must be created as soon as possible.
