# Correction: Association between chronic pain and pre-frailty in Japanese community-dwelling older adults: A cross-sectional study

**DOI:** 10.1371/journal.pone.0261597

**Published:** 2021-12-14

**Authors:** Ryota Imai, Masakazu Imaoka, Hidetoshi Nakao, Mitsumasa Hida, Fumie Tazaki, Tomoko Omizu, Tomoya Ishigaki, Misa Nakamura

There is an error in the fourth sentence of the “The characteristics of the chronic pain and non-chronic pain subjects” subsection of the Results section. The correct sentence is: Compared to the chronic pain group, the non-chronic pain group showed significantly poorer scores for pain intensity, rumination, PCS total score, rumination, helplessness, magnification, CSI and GDS15 score (Table 1).
